# 3-(3-Fluoro­benz­yl)-1*H*-isochromen-1-one

**DOI:** 10.1107/S1600536808035575

**Published:** 2008-11-08

**Authors:** Tariq Mahmood Babar, Ghulam Qadeer, Obaid-ur-Rahman Abid, Nasim Hassan Rama, Ales Ruzicka

**Affiliations:** a Department of Chemistry, Quaid-i-Azam University, Islamabad 45320, Pakistan; b Department of General and Inorganic Chemistry, Faculty of Chemical Technology, University of Pardubice, Nam. Cs. Legii’ 565, 53210 Pardubice, Czech Republic

## Abstract

The asymmetric unit of the title compound, C_16_H_11_FO_2_, contains two independent mol­ecules. The isochromene ring systems are planar and are oriented with respect to the fluoro­benzene rings at dihedral angles of 87.15 (3) and 87.85 (3)° in the two mol­ecules.

## Related literature

For general background, see: Barry (1964 ▶); Hill (1986 ▶); Canedo *et al.* (1997 ▶); Whyte *et al.* (1996 ▶). For a related structure, see: Abid *et al.* (2006 ▶). For bond-length data, see: Allen *et al.* (1987 ▶).
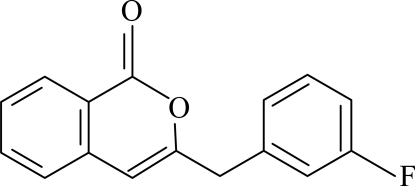


## Experimental

### 

#### Crystal data

C_16_H_11_FO_2_
*M* *_r_* = 254.25
Triclinic, 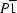

*a* = 7.0130 (7) Å
*b* = 11.7570 (9) Å
*c* = 15.8070 (7) Å
α = 97.515 (6)°
β = 100.520 (4)°
γ = 105.397 (7)°
*V* = 1213.12 (16) Å^3^
*Z* = 4
Mo *K*α radiation
μ = 0.10 mm^−1^
*T* = 150 (1) K
0.38 × 0.24 × 0.22 mm

#### Data collection

Bruker–Nonius KappaCCD area-detector diffractometer
Absorption correction: Gaussian (Coppens, 1970 ▶) *T*_min_ = 0.925, *T*_max_ = 0.961
19724 measured reflections
5458 independent reflections
3741 reflections with *I* > 2σ(*I*)
*R*_int_ = 0.103

#### Refinement

*R*[*F* ^2^ > 2σ(*F* ^2^)] = 0.074
*wR*(*F* ^2^) = 0.218
*S* = 1.15
5458 reflections
343 parameters
H-atom parameters constrained
Δρ_max_ = 1.11 e Å^−3^
Δρ_min_ = −0.33 e Å^−3^

### 

Data collection: *COLLECT* (Hooft, 1998 ▶); cell refinement: *COLLECT* and *DENZO* (Otwinowski & Minor, 1997 ▶); data reduction: *COLLECT* and *DENZO*; program(s) used to solve structure: *SIR92* (Altomare *et al.*, 1994 ▶); program(s) used to refine structure: *SHELXL97* (Sheldrick, 2008 ▶); molecular graphics: *PLATON* (Spek, 2003 ▶); software used to prepare material for publication: *SHELXL97*.

## Supplementary Material

Crystal structure: contains datablocks I, global. DOI: 10.1107/S1600536808035575/hk2565sup1.cif

Structure factors: contains datablocks I. DOI: 10.1107/S1600536808035575/hk2565Isup2.hkl

Additional supplementary materials:  crystallographic information; 3D view; checkCIF report

## Figures and Tables

**Fig. 1.**
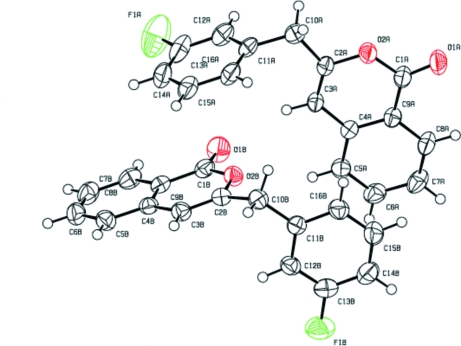
The molecular structure of the title molecule, with the atom-numbering scheme. Displacement ellipsoids are drawn at the 30% probability level.

## supplementary crystallographic information

### Comment

Isocoumarins are secondary metabolites derived from the acetate pathway and are structurally related to the coumarins, but with an inverted lactone ring (Hill, 1986). They are produced by microorganisms, insects and some higher plants, and have a wide range of biological activities, including antitumoral, antileucemic, antiviral and antimicrobial (Hill, 1986; Canedo *et al.*,  1997; Whyte *et al.*,  1996). Isocoumarins (Barry, 1964) are also useful intermediates in the synthesis of a variety of important compounds including some isoquinoline alkaloids. In view of their natural occurrence, biological activities and utility as synthetic intermediates, we have synthesized the title compound, and reported herein its crystal structure.

The asymmetric unit of the title compound contains two crystallographically independent molecules of similar geometry (Fig 1). The bond lengths (Allen *et al.*,  1987) and angles are within normal ranges and comparable with 3-(2-chlorobenzyl)isocoumarin (Abid *et al.*,  2006). Rings A (C1A-C4A/C9A/O2A), B (C4A-C9A), C (C11A-C16A) and D (C1B-C4B/C9B/O2B), E (C4B-C9B), F (C11B-C16B) are, of course, planar and dihedral angles between them are A/B = 1.44 (3)°, A/C = 87.50 (3)°, B/C = 86.91 (4)° and D/E = 0.46 (3)°, D/F = 88.10 (3)°, E/F = 87.65 (3)°.

### Experimental

A mixture of 3-fluorophenylacetic acid (5 g, 32 mmol) and thionyl chloride (2.94 ml, 34 mmol) was heated for 30 min in the presence of a few drops of DMF under reflux at 343 K to give 2-(3-fluorophenyl)acetyl chloride. Completion of reaction was indicated by the disappearance of gas evolution. Removal of excess thionyl chloride was carried out under reduced pressure to afford 2-(3-fluorophenyl)acetyl chloride. Homophthalic acid (1.3 g, 7.2 mmol) was then added and the solution was refluxed for 4 h at 473 K with stirring. The reaction mixture was extracted with ethyl acetate (3 × 100 ml), and an aqueous solution of sodium carbonate (5%, 200 ml) was added to remove the unreacted homophthalic acid. The organic layer was separated, concentrated and chromatographed on silica gel using petroleum ether (313-353 K fractions) as eluent to afford the title compound (yield; 72%, m.p. 447-448 K). Single crystals suitable for X-ray analysis were obtained by slow evaporation of an ethyl acetate solution.

### Refinement

H atoms were positioned geometrically, with C-H = 0.93 and 0.97 Å for aromatic and methylene H, respectively, and constrained to ride on their parent atoms with U_iso_(H) = 1.2U_eq_(C).

### Crystal data

**Table:**

C_16_H_11_FO_2_	*Z* = 4
*M**_r_* = 254.25	*F*_000_ = 528
Triclinic, *P*1	*D*_x_ = 1.392 Mg m^−^^3^
Hall symbol: -P 1	Melting point: 447(1) K
*a* = 7.0130 (7) Å	Mo *K*α radiation λ = 0.71073 Å
*b* = 11.7570 (9) Å	Cell parameters from 19831 reflections
*c* = 15.8070 (7) Å	θ = 1–27.5º
α = 97.515 (6)º	µ = 0.10 mm^−^^1^
β = 100.520 (4)º	*T* = 150 (1) K
γ = 105.397 (7)º	Block, colorless
*V* = 1213.12 (16) Å^3^	0.38 × 0.24 × 0.22 mm

### Data collection

**Table:**

Bruker–Nonius KappaCCD area-detector diffractometer	5458 independent reflections
Radiation source: fine-focus sealed tube	3741 reflections with *I* > 2σ(*I*)
Monochromator: graphite	*R*_int_ = 0.103
Detector resolution: 9.091 pixels mm^-1^	θ_max_ = 27.5º
*T* = 150(1) K	θ_min_ = 1.3º
φ and ω scans	*h* = −9→9
Absorption correction: Gaussian(Coppens, 1970)	*k* = −15→15
*T*_min_ = 0.925, *T*_max_ = 0.961	*l* = −20→20
19724 measured reflections	

### Refinement

**Table:**

Refinement on *F*^2^	Secondary atom site location: difference Fourier map
Least-squares matrix: full	Hydrogen site location: inferred from neighbouring sites
*R*[*F*^2^ > 2σ(*F*^2^)] = 0.074	H-atom parameters constrained
*wR*(*F*^2^) = 0.218	*w* = 1/[σ^2^(*F*_o_^2^) + (0.0736*P*)^2^ + 0.9938*P*] where *P* = (*F*_o_^2^ + 2*F*_c_^2^)/3
*S* = 1.15	(Δ/σ)_max_ < 0.001
5458 reflections	Δρ_max_ = 1.11 e Å^−^^3^
343 parameters	Δρ_min_ = −0.33 e Å^−^^3^
Primary atom site location: structure-invariant direct methods	Extinction correction: none

### Special details

**Table:**

Geometry. All e.s.d.'s (except the e.s.d. in the dihedral angle between two l.s. planes) are estimated using the full covariance matrix. The cell e.s.d.'s are taken into account individually in the estimation of e.s.d.'s in distances, angles and torsion angles; correlations between e.s.d.'s in cell parameters are only used when they are defined by crystal symmetry. An approximate (isotropic) treatment of cell e.s.d.'s is used for estimating e.s.d.'s involving l.s. planes.
Refinement. Refinement of *F*^2^ against ALL reflections. The weighted *R*-factor *wR* and goodness of fit *S* are based on *F*^2^, conventional *R*-factors *R* are based on *F*, with *F* set to zero for negative *F*^2^. The threshold expression of *F*^2^ > σ(*F*^2^) is used only for calculating *R*-factors(gt) *etc*. and is not relevant to the choice of reflections for refinement. *R*-factors based on *F*^2^ are statistically about twice as large as those based on *F*, and *R*- factors based on ALL data will be even larger.

### Fractional atomic coordinates and isotropic or equivalent isotropic displacement parameters (Å^2^)

**Table:**

	*x*	*y*	*z*	*U*_iso_*/*U*_eq_	
F1A	−0.3150 (4)	−0.1110 (3)	0.3167 (2)	0.1171 (11)	
O1A	0.3057 (4)	0.66409 (18)	0.51835 (14)	0.0554 (6)	
O2A	0.2277 (3)	0.46688 (17)	0.49561 (12)	0.0445 (5)	
C1A	0.2626 (5)	0.5733 (2)	0.46489 (18)	0.0396 (6)	
C2A	0.1748 (4)	0.3568 (2)	0.44071 (18)	0.0366 (6)	
C3A	0.1539 (4)	0.3464 (2)	0.35504 (17)	0.0327 (6)	
H3A	0.1165	0.2708	0.3196	0.039*	
C4A	0.1886 (4)	0.4520 (2)	0.31613 (16)	0.0307 (5)	
C5A	0.1696 (4)	0.4466 (3)	0.22617 (17)	0.0376 (6)	
H5A	0.1304	0.3727	0.1884	0.045*	
C6A	0.2087 (5)	0.5508 (3)	0.19368 (19)	0.0448 (7)	
H6A	0.1947	0.5469	0.1336	0.054*	
C7A	0.2686 (5)	0.6617 (3)	0.2486 (2)	0.0494 (8)	
H7A	0.2961	0.7316	0.2255	0.059*	
C8A	0.2869 (5)	0.6691 (3)	0.3371 (2)	0.0438 (7)	
H8A	0.3264	0.7436	0.3741	0.053*	
C9A	0.2464 (4)	0.5642 (2)	0.37126 (17)	0.0336 (6)	
C10A	0.1492 (5)	0.2591 (3)	0.49354 (19)	0.0477 (8)	
H10AA	0.0384	0.2597	0.5223	0.057*	
H10AB	0.2721	0.2754	0.5387	0.057*	
C11A	0.1064 (5)	0.1367 (2)	0.43876 (19)	0.0415 (7)	
C12A	−0.0899 (5)	0.0666 (3)	0.4031 (2)	0.0500 (8)	
H12A	−0.1986	0.0926	0.4142	0.060*	
C13A	−0.1233 (6)	−0.0442 (3)	0.3506 (2)	0.0597 (9)	
C14A	0.0309 (6)	−0.0860 (3)	0.3330 (2)	0.0605 (9)	
H14A	0.0045	−0.1603	0.2972	0.073*	
C15A	0.2262 (6)	−0.0157 (3)	0.3690 (2)	0.0582 (9)	
H15A	0.3335	−0.0431	0.3576	0.070*	
C16A	0.2644 (5)	0.0946 (3)	0.4218 (2)	0.0486 (8)	
H16A	0.3979	0.1410	0.4462	0.058*	
F1B	0.6734 (4)	0.4452 (2)	0.02309 (13)	0.0755 (7)	
O1B	−0.0906 (3)	0.1067 (2)	0.15717 (17)	0.0560 (6)	
O2B	0.2406 (3)	0.14988 (17)	0.19855 (12)	0.0379 (5)	
C1B	0.0550 (4)	0.0724 (3)	0.15393 (19)	0.0377 (6)	
C2B	0.4190 (4)	0.1206 (2)	0.20150 (17)	0.0338 (6)	
C3B	0.4207 (4)	0.0158 (2)	0.15905 (17)	0.0362 (6)	
H3B	0.5434	−0.0013	0.1613	0.043*	
C4B	0.2357 (4)	−0.0713 (2)	0.10937 (16)	0.0335 (6)	
C5B	0.2287 (5)	−0.1833 (3)	0.06398 (18)	0.0414 (7)	
H5B	0.3484	−0.2037	0.0651	0.050*	
C6B	0.0472 (5)	−0.2627 (3)	0.01779 (19)	0.0479 (8)	
H6B	0.0445	−0.3373	−0.0115	0.057*	
C7B	−0.1311 (5)	−0.2330 (3)	0.0146 (2)	0.0486 (8)	
H7B	−0.2535	−0.2871	−0.0174	0.058*	
C8B	−0.1287 (4)	−0.1243 (3)	0.0585 (2)	0.0448 (7)	
H8B	−0.2490	−0.1044	0.0564	0.054*	
C9B	0.0545 (4)	−0.0429 (2)	0.10674 (17)	0.0346 (6)	
C10B	0.5958 (4)	0.2185 (3)	0.25670 (17)	0.0385 (6)	
H10BA	0.7166	0.1925	0.2602	0.046*	
H10BB	0.5740	0.2328	0.3155	0.046*	
C11B	0.6324 (4)	0.3355 (2)	0.22315 (17)	0.0341 (6)	
C12B	0.6343 (4)	0.3367 (3)	0.13607 (18)	0.0393 (6)	
H12B	0.6121	0.2656	0.0971	0.047*	
C13B	0.6700 (5)	0.4442 (3)	0.10830 (19)	0.0452 (7)	
C14B	0.7068 (5)	0.5520 (3)	0.1630 (2)	0.0512 (8)	
H14B	0.7312	0.6238	0.1423	0.061*	
C15B	0.7077 (5)	0.5502 (3)	0.2497 (2)	0.0507 (8)	
H15B	0.7325	0.6218	0.2886	0.061*	
C16B	0.6707 (4)	0.4437 (3)	0.28008 (19)	0.0418 (7)	
H16B	0.6724	0.4442	0.3391	0.050*	

### Atomic displacement parameters (Å^2^)

**Table:**

	*U*^11^	*U*^22^	*U*^33^	*U*^12^	*U*^13^	*U*^23^
F1A	0.0800 (19)	0.0779 (18)	0.155 (3)	−0.0105 (14)	−0.0160 (18)	0.0182 (18)
O1A	0.0836 (17)	0.0300 (11)	0.0420 (12)	0.0070 (11)	0.0081 (11)	−0.0009 (9)
O2A	0.0694 (14)	0.0310 (10)	0.0299 (10)	0.0109 (9)	0.0095 (9)	0.0056 (8)
C1A	0.0471 (17)	0.0285 (14)	0.0389 (15)	0.0074 (12)	0.0048 (12)	0.0061 (11)
C2A	0.0453 (16)	0.0264 (13)	0.0390 (14)	0.0099 (11)	0.0112 (12)	0.0083 (11)
C3A	0.0368 (14)	0.0248 (12)	0.0353 (13)	0.0085 (10)	0.0060 (11)	0.0055 (10)
C4A	0.0292 (13)	0.0292 (13)	0.0331 (13)	0.0076 (10)	0.0061 (10)	0.0079 (10)
C5A	0.0428 (16)	0.0361 (15)	0.0323 (13)	0.0111 (12)	0.0054 (11)	0.0072 (11)
C6A	0.0510 (18)	0.0479 (17)	0.0349 (14)	0.0116 (14)	0.0076 (12)	0.0157 (13)
C7A	0.0557 (19)	0.0398 (16)	0.0495 (17)	0.0071 (14)	0.0049 (14)	0.0221 (14)
C8A	0.0499 (17)	0.0276 (14)	0.0475 (16)	0.0048 (12)	0.0033 (13)	0.0099 (12)
C9A	0.0333 (14)	0.0295 (13)	0.0348 (13)	0.0070 (11)	0.0029 (10)	0.0066 (10)
C10A	0.071 (2)	0.0397 (16)	0.0385 (15)	0.0171 (15)	0.0179 (14)	0.0174 (13)
C11A	0.0545 (18)	0.0313 (14)	0.0437 (15)	0.0119 (13)	0.0152 (13)	0.0207 (12)
C12A	0.0550 (19)	0.0431 (17)	0.0571 (19)	0.0150 (15)	0.0140 (15)	0.0250 (15)
C13A	0.059 (2)	0.0387 (18)	0.067 (2)	−0.0041 (16)	−0.0014 (17)	0.0204 (16)
C14A	0.087 (3)	0.0299 (16)	0.062 (2)	0.0090 (17)	0.0189 (19)	0.0120 (15)
C15A	0.073 (2)	0.0362 (17)	0.075 (2)	0.0171 (16)	0.0324 (19)	0.0210 (16)
C16A	0.0538 (19)	0.0339 (15)	0.0593 (19)	0.0079 (14)	0.0165 (15)	0.0185 (14)
F1B	0.1003 (18)	0.0857 (16)	0.0531 (12)	0.0332 (14)	0.0296 (11)	0.0283 (11)
O1B	0.0363 (12)	0.0522 (13)	0.0832 (17)	0.0170 (10)	0.0194 (11)	0.0088 (12)
O2B	0.0337 (10)	0.0353 (10)	0.0443 (11)	0.0099 (8)	0.0113 (8)	0.0032 (8)
C1B	0.0322 (14)	0.0369 (15)	0.0462 (15)	0.0080 (12)	0.0138 (12)	0.0133 (12)
C2B	0.0331 (14)	0.0374 (14)	0.0325 (13)	0.0106 (11)	0.0095 (10)	0.0089 (11)
C3B	0.0317 (14)	0.0392 (15)	0.0389 (14)	0.0117 (11)	0.0104 (11)	0.0060 (11)
C4B	0.0355 (14)	0.0353 (14)	0.0306 (12)	0.0092 (11)	0.0100 (10)	0.0091 (11)
C5B	0.0475 (17)	0.0401 (16)	0.0363 (14)	0.0137 (13)	0.0092 (12)	0.0051 (12)
C6B	0.060 (2)	0.0355 (15)	0.0403 (16)	0.0058 (14)	0.0082 (14)	0.0019 (12)
C7B	0.0432 (17)	0.0437 (17)	0.0461 (17)	−0.0032 (13)	0.0020 (13)	0.0082 (14)
C8B	0.0328 (15)	0.0486 (18)	0.0495 (17)	0.0044 (13)	0.0084 (12)	0.0148 (14)
C9B	0.0343 (14)	0.0355 (14)	0.0347 (13)	0.0070 (11)	0.0105 (11)	0.0126 (11)
C10B	0.0385 (15)	0.0399 (15)	0.0317 (13)	0.0077 (12)	0.0032 (11)	0.0035 (11)
C11B	0.0252 (13)	0.0374 (14)	0.0348 (13)	0.0043 (11)	0.0050 (10)	0.0032 (11)
C12B	0.0385 (15)	0.0423 (16)	0.0335 (14)	0.0083 (12)	0.0087 (11)	0.0012 (12)
C13B	0.0427 (16)	0.0563 (19)	0.0384 (15)	0.0133 (14)	0.0125 (12)	0.0137 (14)
C14B	0.0471 (18)	0.0441 (18)	0.065 (2)	0.0102 (14)	0.0188 (15)	0.0183 (15)
C15B	0.0509 (18)	0.0377 (16)	0.0570 (19)	0.0054 (14)	0.0142 (15)	0.0003 (14)
C16B	0.0384 (15)	0.0439 (16)	0.0353 (14)	0.0045 (12)	0.0060 (11)	0.0000 (12)

### Geometric parameters (Å, °)

**Table:**

O1A—C1A	1.202 (3)	O2B—C1B	1.374 (3)
O2A—C1A	1.378 (3)	O2B—C2B	1.378 (3)
O2A—C2A	1.378 (3)	C2B—C3B	1.328 (4)
C2A—C3A	1.321 (4)	C2B—C10B	1.486 (4)
C2A—C10A	1.498 (4)	C3B—H3B	0.9299
C3A—H3A	0.9301	C4B—C5B	1.398 (4)
C4A—C3A	1.440 (4)	C4B—C3B	1.436 (4)
C4A—C9A	1.397 (4)	C5B—C6B	1.370 (4)
C5A—C4A	1.395 (4)	C5B—H5B	0.9300
C5A—C6A	1.370 (4)	C6B—H6B	0.9300
C5A—H5A	0.9300	C7B—C6B	1.379 (5)
C6A—C7A	1.382 (4)	C7B—H7B	0.9300
C6A—H6A	0.9300	C8B—C7B	1.367 (4)
C7A—H7A	0.9300	C8B—H8B	0.9300
C8A—C7A	1.370 (4)	C9B—C1B	1.458 (4)
C8A—H8A	0.9300	C9B—C4B	1.393 (4)
C9A—C1A	1.451 (4)	C9B—C8B	1.395 (4)
C9A—C8A	1.391 (4)	C10B—H10BA	0.9700
C10A—H10AA	0.9701	C10B—H10BB	0.9701
C10A—H10AB	0.9700	C11B—C12B	1.381 (4)
C11A—C10A	1.504 (4)	C11B—C16B	1.390 (4)
C11A—C12A	1.373 (5)	C11B—C10B	1.513 (4)
C11A—C16A	1.383 (4)	C12B—H12B	0.9300
C12A—C13A	1.386 (5)	C13B—C12B	1.368 (4)
C12A—H12A	0.9300	C13B—C14B	1.370 (4)
C13A—F1A	1.333 (4)	C14B—H14B	0.9301
C14A—C13A	1.359 (5)	C15B—C14B	1.373 (5)
C14A—C15A	1.368 (5)	C15B—C16B	1.377 (4)
C14A—H14A	0.9300	C15B—H15B	0.9299
C15A—H15A	0.9300	C16A—C15A	1.379 (4)
F1B—C13B	1.353 (3)	C16A—H16A	0.9300
O1B—C1B	1.200 (3)	C16B—H16B	0.9300
			
C2A—O2A—C1A	122.3 (2)	C1B—O2B—C2B	122.5 (2)
O1A—C1A—O2A	116.8 (3)	O1B—C1B—O2B	116.7 (3)
O1A—C1A—C9A	126.6 (3)	O1B—C1B—C9B	126.4 (3)
O2A—C1A—C9A	116.6 (2)	O2B—C1B—C9B	116.9 (2)
C3A—C2A—O2A	122.1 (2)	C3B—C2B—O2B	121.2 (2)
C3A—C2A—C10A	128.3 (2)	C3B—C2B—C10B	127.3 (3)
O2A—C2A—C10A	109.6 (2)	O2B—C2B—C10B	111.4 (2)
C2A—C3A—C4A	120.2 (2)	C2B—C3B—C4B	120.9 (2)
C2A—C3A—H3A	120.1	C2B—C3B—H3B	119.6
C4A—C3A—H3A	119.8	C4B—C3B—H3B	119.5
C5A—C4A—C9A	119.0 (2)	C9B—C4B—C5B	118.5 (3)
C5A—C4A—C3A	122.7 (2)	C9B—C4B—C3B	118.2 (2)
C9A—C4A—C3A	118.3 (2)	C5B—C4B—C3B	123.2 (2)
C6A—C5A—C4A	119.7 (3)	C6B—C5B—C4B	120.5 (3)
C6A—C5A—H5A	120.1	C6B—C5B—H5B	119.9
C4A—C5A—H5A	120.2	C4B—C5B—H5B	119.6
C5A—C6A—C7A	121.1 (3)	C5B—C6B—C7B	120.5 (3)
C5A—C6A—H6A	119.4	C5B—C6B—H6B	119.6
C7A—C6A—H6A	119.4	C7B—C6B—H6B	119.9
C8A—C7A—C6A	120.1 (3)	C8B—C7B—C6B	120.2 (3)
C8A—C7A—H7A	119.9	C8B—C7B—H7B	119.8
C6A—C7A—H7A	120.0	C6B—C7B—H7B	120.0
C7A—C8A—C9A	119.6 (3)	C7B—C8B—C9B	120.1 (3)
C7A—C8A—H8A	120.2	C7B—C8B—H8B	120.2
C9A—C8A—H8A	120.2	C9B—C8B—H8B	119.8
C8A—C9A—C4A	120.5 (2)	C4B—C9B—C8B	120.2 (3)
C8A—C9A—C1A	119.0 (2)	C4B—C9B—C1B	120.2 (2)
C4A—C9A—C1A	120.5 (2)	C8B—C9B—C1B	119.6 (3)
C2A—C10A—C11A	112.7 (2)	C2B—C10B—C11B	113.9 (2)
C2A—C10A—H10AA	109.2	C2B—C10B—H10BA	108.9
C11A—C10A—H10AA	109.1	C11B—C10B—H10BA	108.8
C2A—C10A—H10AB	108.9	C2B—C10B—H10BB	108.7
C11A—C10A—H10AB	109.0	C11B—C10B—H10BB	108.7
H10AA—C10A—H10AB	107.8	H10BA—C10B—H10BB	107.6
C12A—C11A—C16A	119.1 (3)	C12B—C11B—C16B	119.0 (3)
C12A—C11A—C10A	120.5 (3)	C12B—C11B—C10B	120.5 (2)
C16A—C11A—C10A	120.4 (3)	C16B—C11B—C10B	120.5 (2)
C11A—C12A—C13A	118.9 (3)	C13B—C12B—C11B	118.9 (3)
C11A—C12A—H12A	120.6	C13B—C12B—H12B	120.6
C13A—C12A—H12A	120.5	C11B—C12B—H12B	120.4
F1A—C13A—C14A	119.6 (4)	F1B—C13B—C12B	118.7 (3)
F1A—C13A—C12A	117.9 (4)	F1B—C13B—C14B	118.0 (3)
C14A—C13A—C12A	122.5 (3)	C12B—C13B—C14B	123.2 (3)
C13A—C14A—C15A	118.3 (3)	C13B—C14B—C15B	117.5 (3)
C13A—C14A—H14A	120.8	C13B—C14B—H14B	121.3
C15A—C14A—H14A	120.8	C15B—C14B—H14B	121.2
C14A—C15A—C16A	120.6 (3)	C14B—C15B—C16B	121.0 (3)
C14A—C15A—H15A	119.4	C14B—C15B—H15B	119.7
C16A—C15A—H15A	120.0	C16B—C15B—H15B	119.3
C15A—C16A—C11A	120.6 (3)	C15B—C16B—C11B	120.4 (3)
C15A—C16A—H16A	119.6	C15B—C16B—H16B	119.9
C11A—C16A—H16A	119.7	C11B—C16B—H16B	119.8

## References

[bb1] Abid, O., Rama, N. H., Qadeer, G., Khan, G. S. & Lu, X.-M. (2006). *Acta Cryst.* E**62**, o2895–o2896.

[bb2] Allen, F. H., Kennard, O., Watson, D. G., Brammer, L., Orpen, A. G. & Taylor, R. (1987). *J. Chem. Soc. Perkin Trans. 2*, pp. S1–19.

[bb3] Altomare, A., Cascarano, G., Giacovazzo, C., Guagliardi, A., Burla, M. C., Polidori, G. & Camalli, M. (1994). *J. Appl. Cryst.* **27**, 435.

[bb4] Barry, R. D. (1964). *Chem. Rev.* **64**, 229–260.

[bb5] Canedo, L. M., Puents, J. L. F. & Baz, J. P. (1997). *J. Antibiot.* **50**, 175–176.

[bb6] Coppens, P. (1970). *Crystallographic Computing*, edited by F. R. Ahmed, pp. 255–265. Copenhagen: Munksgaard.

[bb7] Hill, R. A. (1986). *Fortschr. Chem. Org. Naturst* **49**, 1–78.

[bb8] Hooft, R. W. W. (1998). *COLLECT* Nonius BV, Delft, The Netherlands.

[bb10] Otwinowski, Z. & Minor, W. (1997). *Methods in Enzymology*, Vol. 276, *Macromolecular Crystallography*, Part A, edited by C. W. Carter Jr & R. M. Sweet, pp. 307–326. New York: Academic Press.

[bb11] Sheldrick, G. M. (2008). *Acta Cryst.* A**64**, 112–122. 10.1107/S0108767307043930 18156677

[bb12] Spek, A. L. (2003). *J. Appl. Cryst.* **36**, 7–13.

[bb13] Whyte, A. C., Gloer, J. B., Scott, J. A. & Malloch, D. (1996). J*. Nat. Prod.* **59**, 765–769. 10.1021/np9603232 8792624

